# Activation of mTORC1 by Free Fatty Acids Suppresses LAMP2 and Autophagy Function via ER Stress in Alcohol-Related Liver Disease

**DOI:** 10.3390/cells10102730

**Published:** 2021-10-13

**Authors:** Wei Guo, Wei Zhong, Liuyi Hao, Xinguo Sun, Zhanxiang Zhou

**Affiliations:** 1 Centers for Translational Biomedical Research, University of North Carolina at Greensboro, North Carolina Research Campus, Kannapolis, NC 28081, USA; w_guo2@uncg.edu (W.G.); w_zhong@uncg.edu (W.Z.); l_hao@uncg.edu (L.H.); x_sun4@uncg.edu (X.S.); 2 Department of Nutrition, University of North Carolina at Greensboro, North Carolina Research Campus, Kannapolis, NC 28081, USA

**Keywords:** mTORC1, free fatty acid, ER stress, LAMP2, inflammation, alcohol-related liver disease

## Abstract

Alcohol-related liver disease (ALD) is characterized by accumulation of hepatic free fatty acids (FFAs) and liver injury. The present study aimed to investigate if mechanistic target of rapamycin complex 1 (mTORC1) plays a role in FFA-induced organelle dysfunction, thereby contributing to the development of ALD. Cell studies were conducted to define the causal role and underlying mechanism of FFA-activated mTORC1 signaling in hepatocellular cell injury. C57BL/6J wild-type mice were subjected to chronic alcohol feeding with or without rapamycin to inhibit mTORC1 activation. We revealed that palmitic acid (PA)-induced ER stress and suppression of LAMP2 and autophagy flux were mTORC1-dependent as rapamycin reversed such deleterious effects. C/EBP homologous protein (CHOP) was downstream of ATF4 which partially modulated LAMP2. Supplementation with rapamycin to alcohol-fed mice attenuated mTORC1 activation and ER stress, restored LAMP2 protein, and improved autophagy, leading to amelioration of alcohol-induced liver injury. Induction of mTORC1 signaling and CHOP were also detected in the liver of patients with severe alcoholic hepatitis. This study demonstrates that hepatic FFAs play a crucial role in the pathogenesis of ALD by activating mTORC1 signaling, thereby inducing ER stress and suppressing LAMP2-autophagy flux pathway, which represents an important mechanism of FFA-induced hepatocellular injury.

## 1. Introduction

Alcohol use disorder has long been known as a risk factor for disease and preventable death worldwide. Alcohol abuse has been causally linked to the development of more than 60 different medical conditions, including alcohol-related liver disease (ALD), which ranges from alcoholic steatosis, an early and reversible stage of ALD, to irreversible stages such as alcoholic hepatitis and fibrosis, which can progress to cirrhosis and eventually hepatocellular carcinoma [1]. Despite the great efforts that have been made in understanding the underlying mechanisms, there are currently no effective therapies available for the treatment of ALD.

The mechanistic target of rapamycin (mTOR) is an evolutionary conserved protein kinase that forms two structurally and functionally distinct complexes, namely mTOR complex 1 (mTORC1) and mTORC2 in mammals [2]. mTORC1 signaling is involved in the modulation of different cellular processes, such as cell proliferation, autophagy, and metabolism [3]. Aberrant mTORC1 activation has been linked to the pathogenesis of various diseases, including ALD [4,5,6]. Although an earlier study has suggested that mTORC1 plays a role in lipid metabolism and hepatocellular apoptosis in human and animal models of ALD [4], the potential mechanisms of mTORC1 signaling in ALD remain to be elucidated.

Previous studies have suggested that chronic alcohol consumption contributes to the release of free fatty acids (FFAs) from adipose tissue and subsequent increase of hepatic FFA influx and accumulation [7,8]. Recent findings by our group have demonstrated that accumulation of hepatic FFAs, rather than triglyceride (TG)-enriched lipid droplets, activates ER stress pathway, which then downregulates lysosome-associated membrane protein 2 (LAMP2)-mediated autophagy flux and results in alcohol-induced liver injury [9]. Autophagy is a highly conserved catabolic process that targets cellular proteins and organelles for lysosomal degradation, which is crucial for regulating cellular homeostasis [10]. Deregulation of autophagy has been implicated in the pathogenesis of many diseases, including ALD [11,12]. It has also been reported that FFAs, such as palmitic acid, are able to activate mTORC1, which results in reactive oxygen species generation, apoptosis, and metabolic changes in other disease models [13,14,15]. However, it is not clear if FFA-induced ER stress signaling and disruption of LAMP2-meditated autophagy flux is via the activation of mTORC1 pathway in ALD. Therefore, this study was undertaken to investigate whether there is a mechanistic link between FFAs and mTORC1 signaling that contributes to the impairment of autophagy flux in ALD.

## 2. Materials and Methods

### 2.1. Mice

Wild-type (WT) C57BL/6J mice were purchased from the Jackson Laboratory (Bar Harbor, ME, USA). Mice were handled and all experiments were carried out under the guidance of the protocol approved by the North Carolina Research Campus Institutional Animal Care and Use Committee.

### 2.2. Human Liver Samples

Liver explant specimens were collected from deidentified patients with severe alcoholic hepatitis (SAH), and wedge biopsies were collected from donor livers (normal subjects) at Johns Hopkins University with the support of NIAAA-funded Clinical Resource for Alcoholic Hepatitis Investigations (R24AA025017). Donor liver tissue sample collection had been approved by Institutional Review Boards at Johns Hopkins Medical Institutions (IRB00107893, IRB00021325).

### 2.3. Chronic Alcohol Feeding and Treatments

A number of 12-week-old male mice were chronically fed a Lieber–DeCarli liquid diet containing alcohol (alcohol-fed; AF) or an isocaloric control liquid diet (pair-fed; PF) for 8 weeks, as described previously [16]. A potent mTORC1 inhibitor, rapamycin (0.04 mg/mouse/day), was given to the liquid diet-fed WT mice, three times a week, starting from the 6th week in the 8-week feeding experiment. All ingredients used for the liquid diets were obtained from Dyets (Bethlehem, PA) except for ethanol (Sigma-Aldrich, St. Louis, MO, USA).

### 2.4. Western Blot

Protein lysates were extracted from mouse liver, Hepa-1c1c7 cells, and donor liver with lysis buffer containing protease and phosphatase inhibitors at recommended concentration (Sigma-Aldrich). Aliquots containing 30 μg of proteins were loaded to sodium dodecyl sulfate-polyacrylamide gel (SDS-PAGE) and transferred onto polyvinylidene difluoride (PVDF) membranes (Bio-Rad, Hercules, CA, USA). After transfer, membranes were blocked with 4% milk and incubated at 4 °C overnight with the following antibodies: anti-S6, anti-p-S6 (Ser235/236), anti-LAMP1, anti-LAMP2, anti-ATF4, anti-CHOP (Cell Signaling Technology, Denver, MA, USA), anti-LC3II (Novus Biologicals, Littleton, CO, USA), anti-β-actin, and anti-GAPDH (Abcam, Cambridge, MA, USA), respectively. Membranes were subsequently washed and incubated with horseradish peroxidase-conjugated secondary goat anti-mouse IgG or goat anti-rabbit IgG (Thermo Scientific, Rockford, IL, USA). The bound protein complexes were detected with enhanced chemiluminescence (Thermo Fisher Scientific, USA) and quantified by Image J (NIH, Bethesda, MD, USA).

### 2.5. Cell Culture and Treatments

The murine hepatoma cell line, Hepa-1c1c7, was purchased from American Type Culture Collections (Rockville, MD, USA). Cells were cultured in Dulbecco’s Modified Eagle’s Medium (DMEM) containing 10% heat-inactivated fetal bovine serum (FBS), 100 units/mL penicillin, and 100 μg/mL streptomycin (Gibco, Thermo Fisher Scientific, MA, USA). Cells were propagated at a density of 1 × 10^5^ cells/cm^2^ in 10% FBS until reaching 80% confluency. Cells were then treated with palmitic acid (PA) at 100 µmol/L for 24 h. PA was conjugated to bovine serum albumin (BSA) at a ratio of 6.6:1, as previously described [17]. Rapamycin (50nM) and/or tunicamycin (TM, 5 µM) were used for the treatment.

### 2.6. Plasmids Transfection

Hepa-1c1c7 cells were seeded 24 h prior to transfection. After cells reached 80% confluency, CHOP CRISPR/Cas9 KO plasmid (SC-41970, Santa Cruz Biotechnology) or control CRISPR plasmid (SC-437275, Santa Cruz Biotechnology) were transfected to cells following the manufacturer’s instruction. Transfection efficiency was determined by immunoblot analysis.

For the evaluation of autophagy flux, Hepa1c1c7 cells were transfected with pTF-LC3 (plasmid with Tandem Fluorescent tagged LC3, Plasmid ID # 21074) plasmid (Addgene, Cambridge, MA, USA). This plasmid construct comprises the autophagosome marker LC3 tagged with both red fluorescent and green fluorescent protein in tandem (mRFP-GFP-LC3).

### 2.7. Histopathology and Immunohistochemistry

Histopathology staining was performed as previously described [18]. Briefly, mouse liver tissues were fixed in 10% formalin and processed for paraffin embedding. Paraffin sections were cut into 5 μm and processed with hematoxylin and eosin (H&E) staining. LAMP2, CHOP, p-S6 (Ser235/236), and 4-hydroxynonenal (4-HNE) in mouse liver were detected by immunohistochemistry staining. Hydrogen peroxide (3%) was used to deactivate endogenous peroxidases in paraffin-embedded liver sections. A mouse-to-mouse blocking reagent (ScyTek Laboratories, Logan, UT) was used to block the endogenous mouse IgG. Subsequently, tissue sections were incubated with a monoclonal antibody at 4 °C overnight, followed by a 30 min incubation with EnVision^+^ labeled polymer-HRP-conjugated IgG against mouse or rabbit. (DAKO, Carpinteria, CA, USA).

### 2.8. Immunofluorescence

To evaluate hepatic inflammation, cryostat liver sections from control subjects and SAH patients as well as mouse liver were incubated with antimyeloperoxidase (MPO), anti-p-mTORC1 (Ser2448), anti-p-S6 (Ser235/236), or anti-LC3II antibody followed by Alexa Fluor 594-conjugated donkey anti-rat IgG (Jackson ImmunoResearch Laboratories, West Grove, PA). Then, 4′6-diamidino-2-phenylindole (DAPI; Thermo Fisher Scientific) was used to counterstain the nuclei. To determine autophagy flux, accumulation of GFP- and/or RFP-LC3 signals was visualized by fluorescence microscope and quantified according to previous publication [19].

### 2.9. Plasma ALT/AST Levels

Levels of liver injury indicators, such as plasma alanine aminotransferase (ALT) and aspartate aminotransferase (AST), were colorimetrically measured by Infinity ALT Reagent and Infinity AST Reagent (Thermo Scientific, MA, USA), respectively.

### 2.10. RNA Isolation and Real-Time PCR

Total RNA from mouse liver tissue and Hepa-1c1c7 cells was isolated using TRIzol Reagent (Invitrogen, Oregon, USA) according to the manufacturer’s instruction. The complementary DNA (cDNA) was synthesized with the cDNA Synthesis Kit (TaqMan Reverse Transcription Reagents; Thermo Fisher Scientific) and amplified under a 7500 Real-Time PCR System with SYBR Green PCR Supermix Kit (Qiagen). All primers were purchased from Integrated DNA Technologies (Coralville, CA, USA). Data were normalized to RPS17 and measured as relative differences using the 2^−ΔΔCt^ threshold cycle method. Primers used in the analysis were shown as follows:

*Cxcl1*-forward: 5′-CCAGAGCTTGAAGGTGTTGC-3′.

*Cxcl1*-reverse: 5′-AAGCCTCGCGACCATTCTTG-3′.

*Ly6g*-forward: 5′-CCACTCCTCTCTAGGACTTTCA-3′.

*Ly6g*-reverse: 5′-ACCTTGGAATACTGCCTCTTTC-3′.

*RPS17*-forward: 5′-GGAGATCGCCATTATCCCCA-3′.

*RPS17*-reverse: 5′-ATCTCCTTGGTGTCGGGATC-3′.

### 2.11. Quantification of FFAs and TG

Levels of hepatic and cellular FFAs and TG were measured as previously described [16]. Briefly, lipids were extracted using chloroform/methanol (2:1), vacuumed, and redissolved in 5% Triton X-100/methyl alcohol mixture (1:1 vol/vol). Lipid contents were then colorimetrically determined by assay kits (Biovision, Milpitas, CA, USA), as per the manufacturer’s protocol.

### 2.12. Lactate Dehydrogenase (LDH) Assay

Cellular LDH activity was measured using LDH assay kit (Thermo Scientific, MA, USA). Briefly, after each treatment, Hepa-1c1c7 cell culture supernatants were collected, mixed with LDH reaction buffer, and incubated in the dark for 30 min at room temperature, followed by the addition of LDH stop solution. LDH release was then quantified by measuring absorbance at 490 nm and 680 nm to determine LDH activity.

### 2.13. Statistical Analysis

The analyses were performed using SPSS 19.0 software. All experiments were repeated three times to validate results and ensure reliability. All experimental results were analyzed using the independent-samples T-test or one-way analysis of variance (ANOVA) followed by Bonferroni’s multiple comparison. Data were expressed as mean ± standard deviation (SD). In all tests, a *p* value of less than 0.05 was considered statistically significant.

## 3. Results

### 3.1. FFA-induced ER Stress, LAMP2 Reduction, and Impaired Autophagy Flux Is mTORC1-Dependent in Hepatocytes

**Figure 1:**
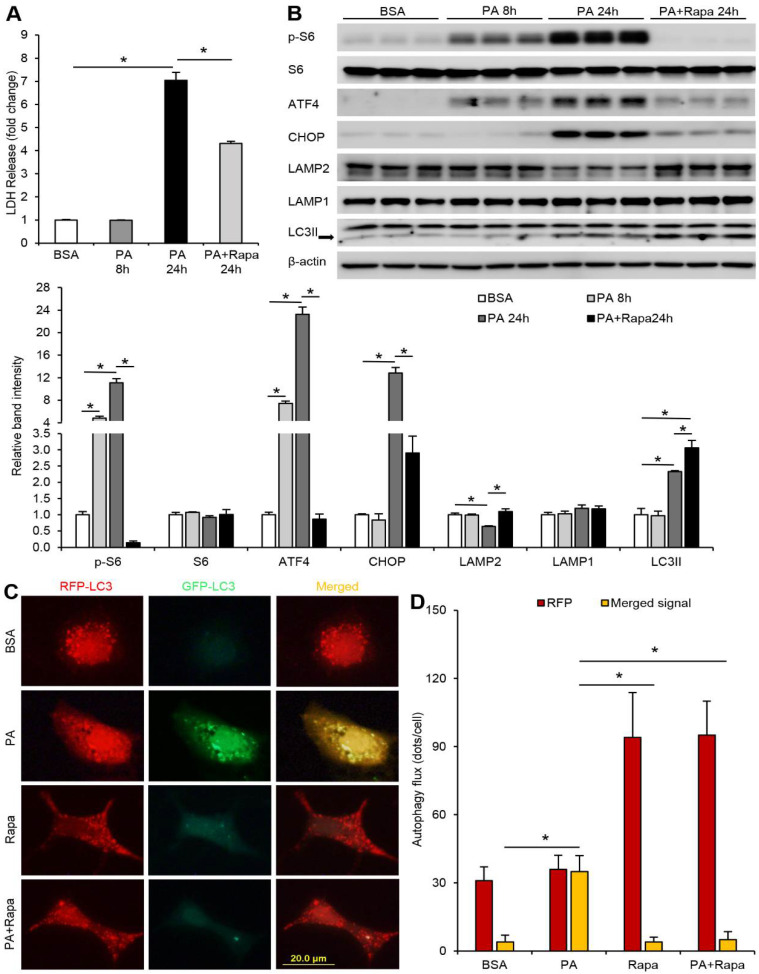
PA-induced LAMP2 reduction, ER stress, and impaired autophagy flux is mTOR-dependent in hepatocytes. Hepa-1c1c7 cells, with or without transfection of tandem fluorescent mRFP-GFP-tagged LC3 plasmid (ptfLC3) were treated with 100 µM palmitic acid (PA) for 24 h in the absence or presence of a potent mTORC1 inhibitor rapamycin (50 nM). (**A**) Cellular LDH levels. (**B**) Immunoblot and quantification analysis of cellular protein levels of p-S6, S6, ATF4, CHOP, LAMP2, LAMP1, and LC3II. (**C**) Autophagy flux was visualized by immunofluorescence (IF) microscopy. (**D**) Quantification of LC3 dots in the cell. Red bars represent RFP-positive dots and yellow bars represent colocalization of RFP and GFP dots when merged. Scale bars, 20 μm. A one-way ANOVA test was performed to calculate the significance of the data (* *p* < 0.05). BSA, bovine serum albumin. PA, palmitic acid.

It has been reported that FFAs induce cell injury via activation ofmTORC1, leading to disruption of autophagy function [13]. We have recently reported that FFAs suppressed LAMP2-mediated autophagy flux via ER stress signaling in ALD [9]. To determine if FFA-induced ER stress, LAMP2 reduction, and autophagy flux blockage are mediated by mTORC1 signaling, Hepa-1c1c7 cells were treated with palmitic acid (PA) in the presence or absence of a specific mTORC1 inhibitor, rapamycin. We found that PA-induced cell injury, as indicated by lactate dehydrogenase (LDH) release, was attenuated by rapamycin (Figure 1A). PA time-dependently increased the phosphorylation of ribosomal protein S6, a well-recognized downstream target of the mTORC1 signaling, which was completely diminished by rapamycin (Figure 1B). Meanwhile, rapamycin abolished PA-induced activating transcription factor 4 (ATF4) and C/EBP homologous protein (CHOP) (Figure 1B). Rapamycin treatment also attenuated PA-mediated reduction of LAMP2 protein without affecting LAMP1 (Figure 1B). Interestingly, we noticed that addition of rapamycin to PA-treated cells led to an even greater accumulation of microtubule-associated protein light chain 3 II (LC3II) compared to PA treatment alone (Figure 1B). LC3II is involved in the autophagy process and its accumulation could indicate impaired autophagy [20]. To address the question of why rapamycin restored LAMP2 but did not attenuate LC3II accumulation, we next performed autophagy flux assay to dissect autophagosome formation and autophagy flux. Hepa-1c1c7 cells were transiently transfected with a tandem-tagged ptfLC3 (mRFP-EGFP-LC3) and treated with PA, rapamycin, or in combination. Virtualization of only mRFP fluorescence signal indicates successful autophagosome–lysosome fusion as the GFP signal is quenched inside the autolysosome with acidic environment. As shown in Figure 1C,D, PA treatment led to an accumulation of both RFP and GFP signals in the cell, which resulted in a yellow signal when merged, suggesting blockage of autophagy flux. Rapamycin alone largely increased the amount of RFP-LC3, while GFP signal was barely visible. Moreover, rapamycin also markedly reduced the accumulation of GFP-LC3 in PA-treated cells, leading to a strong RFP signal when merged, indicating improvement of autophagy flux. These data suggest that FFA-induced ER stress and suppression of LAMP2 is dependent on mTORC1 activation, and inhibition of mTORC1 signaling enhances autophagy flux via upregulation of LAMP2.

### 3.2. mTORC1-Induced ER Stress Targets LAMP2 via ATF4-CHOP Pathway under FFA Treatment in Hepatocytes

**Figure 2:**
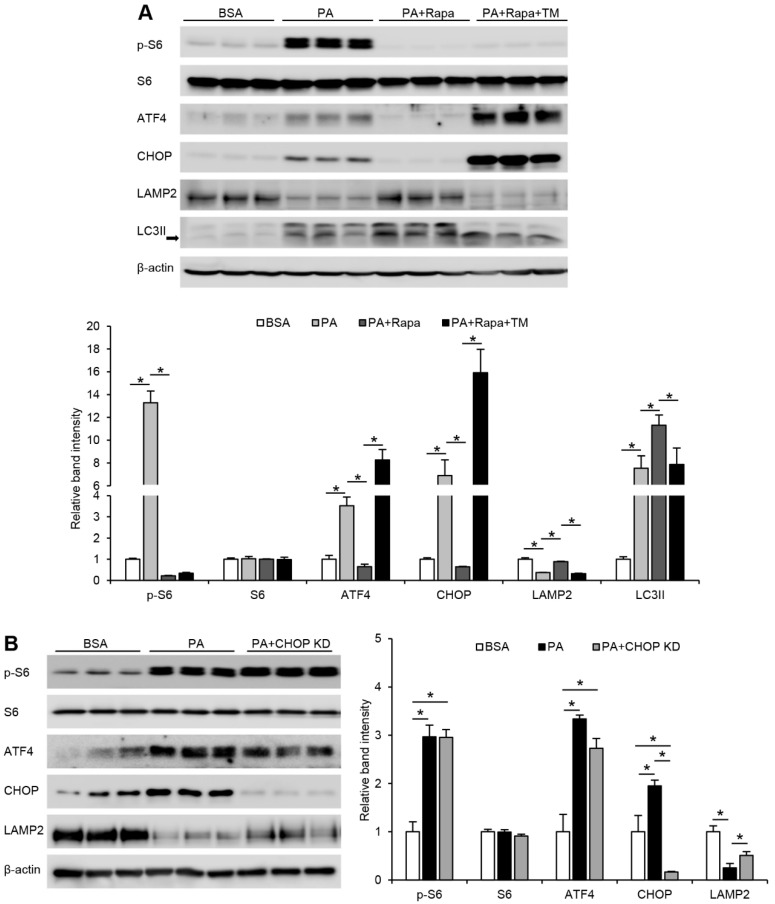
mTORC1-induced ER stress targets LAMP2 via ATF4-CHOP pathway under FFA treatment in hepatocytes. Hepa-1c1c7 cells were treated with PA alone, or in combination with rapamycin in the absence or presence of a potent ER stress inducer, tunicamycin (TM), at 5 µM for 24 h. (**A**) Immunoblot and quantification analysis of cellular protein levels of p-S6, S6, ATF4, CHOP, LAMP2, and LC3II. (**B**) Hepa-1c1c7 cells, with or without transfection of CHOP knockdown CRISPR plasmid, were treated with 100 µM PA for 24 h and immunoblotted for cellular p-S6, S6, ATF4, CHOP, and LAMP2. A one-way ANOVA test was performed to calculate the significance of the data (* *p* < 0.05).

Earlier evidence has suggested that ER stress can also act upstream of mTORC1 in mediating certain cellular pathological changes [21]. To further confirm that FFA-induced ER stress is downstream of mTORC1 signaling which causes the suppression of LAMP2 and accumulation of LC3II, Hepa-1c1c7 cells were treated with PA or PA plus rapamycin in the presence of a potent ER stress inducer, tunicamycin (TM), for 24 h. As shown in Figure 2A, suppressing PA-induced activation of mTORC1 signaling by rapamycin markedly blocked ER stress and restored the level of LAMP2 protein. Addition of TM to PA- and rapamycin-cotreated cells led to an even greater induction of ER stress, while mTORC1 signaling was not reactivated. Rapamycin-rescued LAMP2 protein was inhibited by TM treatment. Previously, we have demonstrated that LAMP2 is a downstream target of ATF4 [9]. It is also well recognized that CHOP is regulated by ATF4 [22]. To elucidate if CHOP is mediating ATF4-induced suppression of LAMP2, we generated a stable CHOP knockdown Hepa-1c1c7 cell line and treated the cells with PA. We found that PA-induced CHOP protein levels were largely abrogated by CHOP knockdown, while PA-suppressed LAMP2 protein levels were partially restored (Figure 2B). In addition, levels of p-S6 protein were not affected by CHOP knockdown. Collectively, these data suggest that FFA-mediated suppression of LAMP2 is downstream of mTORC1-ATF4-CHOP signaling, and modulation of ER stress did not affect upstream mTORC1 activity.

### 3.3. Inhibition of mTORC1 Signaling by Dietary Rapamycin Supplementation Ameliorates Alcohol-Induced ER Stress and Improves Autophagy in Mice

**Figure 3:**
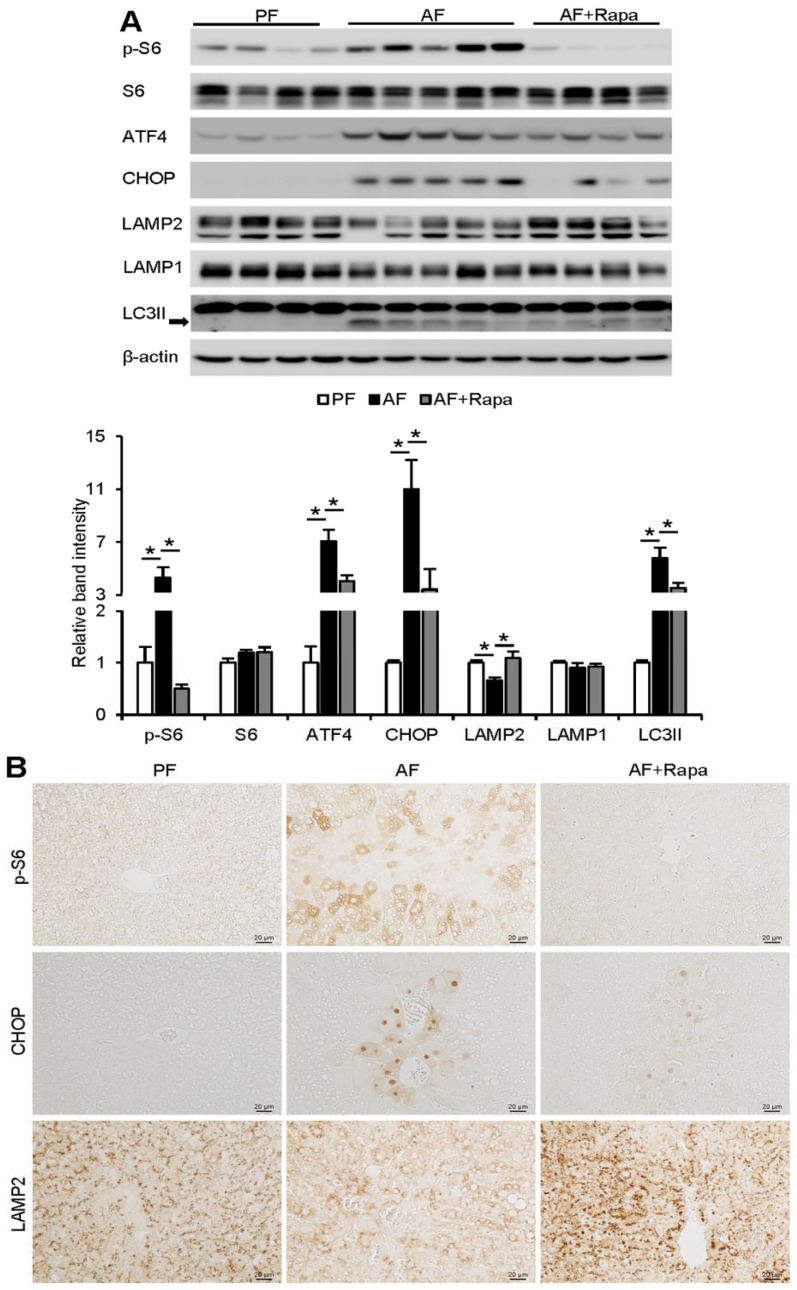
Inhibition of mTORC1 signaling by dietary rapamycin supplementation ameliorates alcohol-induced ER stress and improves autophagy in mice. WT mice were fed a control (PF) or alcohol liquid diet (AF) with or without rapamycin supplementation at 0.04 mg/mouse/day, three times a week, starting from the 6th week in an 8-week feeding experiment. (**A**) Immunoblot and quantification analysis of hepatic protein levels of p-S6, S6, ATF4, CHOP, LAMP2, LAMP1, and LC3II. (**B**) Immunohistochemistry staining of p-S6, CHOP, and LAMP2 on liver tissue sections. Scale bars, 20 μm. A one-way ANOVA test was performed to calculate the significance of the data (* *p* < 0.05).

To further explore whether inhibition of mTOR signaling would inhibit ER stress, upregulate LAMP2, and improve autophagy function, C57BL/6J mice were subjected to chronic alcohol feeding with or without rapamycin supplementation for the last 3 weeks in an 8-week feeding protocol. Alcohol increased the phosphorylation of S6 in the liver, indicating the activation of mTORC1 signaling (Figure 3A). Rapamycin supplementation almost completely blocked alcohol-induced S6 phosphorylation and ameliorated alcohol-induced ER stress, as indicated by decreased levels of ATF4 and CHOP (Figure 3A,B). Alcohol-suppressed LAMP2 protein was rescued by rapamycin (Figure 3A,B). Moreover, rapamycin suppressed alcohol-induced LC3II, suggesting an improvement of autophagy function (Figure 3A).

### 3.4. Inhibition of mTORC1 Signaling by Dietary Rapamycin Supplementation Attenuates Alcohol-Induced Liver Injury in Mice

**Figure 4:**
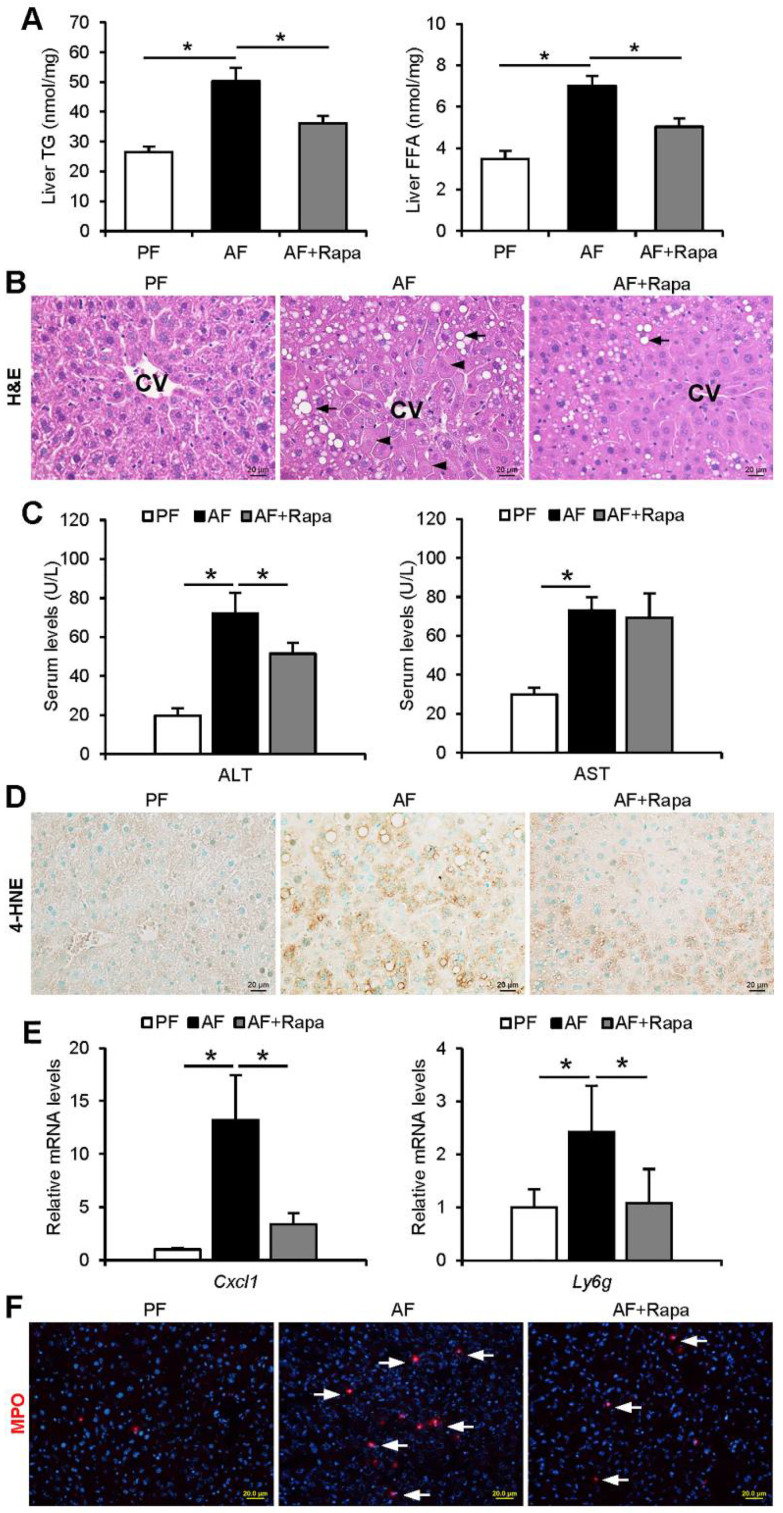
Inhibition of mTOR signaling by dietary rapamycin supplementation attenuates alcohol-induced lipid accumulation and liver injury in mice. WT mice were fed a control (PF) or alcohol liquid diet (AF) with or without rapamycin supplementation at 0.04 mg/mouse/day, three times a week, starting from the 6th week in an 8-week feeding experiment. (**A**) Analysis of hepatic FFA and TG contents (n = 4/group). (**B**) H&E staining of liver tissue sections (n = 4/group). Arrowheads: hepatocyte degeneration. Arrows: hepatic lipid droplets. Scale bars, 20 μm. (**C**) Analysis of serum ALT and AST levels (n = 4/group). (**D**) Immunohistochemistry staining of hepatic 4-HNE on liver tissue sections. Scale bars, 20 μm. (**E**) Hepatic *Cxcl1* and *Ly6g* mRNA expression (n = 4/group). (**F**) Immunofluorescence staining of hepatic MPO positive cells (red) (n = 4/group). Scale bars, 20 μm. Data are shown as means ± SD. A one-way ANOVA test was performed to calculate the significance of the data (* *p* < 0.05).

We next examined the effect of rapamycin supplementation on alcohol-induced liver injury. As illustrated in Figure 4A, alcohol-induced hepatic accumulation of FFAs and TG were both reduced by rapamycin supplementation (Figure 4A,B). Hepatocellular damage was assessed by evaluating serum ALT and AST as well as histopathological changes. Rapamycin supplementation ameliorated alcohol-induced hepatocyte degeneration (Figure 4B), suppressed the elevation of serum ALT level (Figure 4C), and reduced hepatic oxidative stress and inflammation (Figure 4D). Alcohol-induced hepatic inflammation, as indicated by upregulated *Cxcl1* and *Ly6g* mRNA expression (Figure 4E) and infiltrated MPO-positive neutrophils (Figure 4F), was also attenuated by rapamycin supplementation. These data suggest that rapamycin protects against alcohol-induced liver injury by suppressing ER stress signaling and improving autophagy function.

### 3.5. Activation of mTORC1 and Accumulation of CHOP in the Liver of Patients with SAH

**Figure 5:**
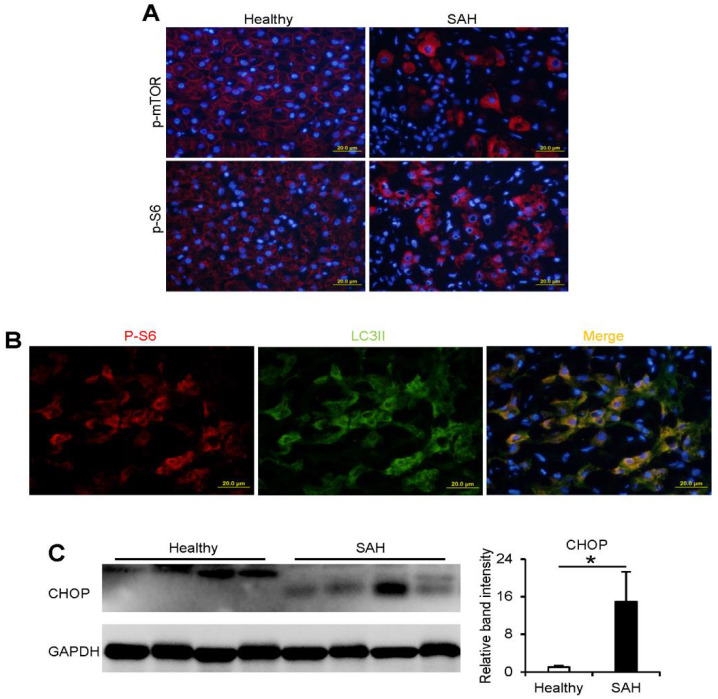
Activation of mTORC1 and accumulation of CHOP in the liver of patients with SAH. (**A**,**B**) IF analysis of p-mTOR, p-S6, and LC3II in the liver of healthy subjects and SAH patients. Scale bars, 20 μm. (**C**) Immunoblot and quantification analysis of CHOP in the liver of healthy subjects and SAH patients. An independent T-test was performed to calculate the significance of the data (* *p* < 0.05).

Our recent publications revealed that the protein levels of ATF4 and LC3II were both increased in the liver of patients with SAH while LAMP2 protein was decreased, linking LAMP2-autophagy to ER stress in the pathogenesis of ALD [9,23]. Here, we further investigated if activation of mTORC1 signaling and CHOP are associated with the pathogenesis of human SAH. As shown in Figure 5A, immunofluorescence analysis revealed that the protein levels of p-mTORC1 and p-S6 were significantly elevated in normal hepatocytes of SAH patients, compared with control subjects. Moreover, the accumulation of cytoplasmic LC3II colocalized with p-S6 in the liver of SAH patients (Figure 5B). In addition, the protein levels of CHOP were significantly increased in the liver of patients with SAH (Figure 5C). These data suggest that our findings about the activation of mTORC1-ATF4-CHOP-LAMP2 signaling in mouse models of ALD is of clinical relevance.

## 4. Discussion

Increased hepatic FFA accumulation is frequently observed in human and animal models of ALD. However, it remains unclear how FFAs incite lipotoxicity and inflammatory responses in the liver. Our cell studies revealed that suppression of LAMP2 and impairment of autophagy flux by PA-induced ER stress is mTORC1-dependent, as inhibition of mTORC1 ameliorated ER stress, restored the protein levels of LAMP2, and improved autophagy flux. We also showed that CHOP is a downstream mediator of ATF4 which plays a partial role in the suppression of LAMP2. Our animal studies further demonstrated that pharmacological inhibition of mTORC1 activation by rapamycin suppressed alcohol-induced ER stress and restored LAMP2 protein and autophagy function, therefore ameliorating alcoholic liver injury. Taken together, our study provides evidence that hepatic FFAs induce lipotoxicity via activation of the mTORC1-ATF4-CHOP pathway, which downregulates LAMP2, thereby impairing autophagy flux and causing subsequent liver damage in ALD.

mTORC1 is a main nutrient-sensing kinase that can be activated by nutrient-related molecules such as glucose, insulin, and amino acids, leading to various cellular processes via different regulatory mechanisms [2,3]. Abnormal mTORC1 activation has been implicated in nonalcoholic fatty liver disease [24,25]. However, under the condition of chronic alcohol feeding, mechanisms involved in the activation of mTORC1 pathway as well as its downstream cellular responses remain to be elucidated. A recent study by Chen et al. showed that alcohol feeding causes the suppression of sirtuin 1 and DEP-domain-containing mTOR-interacting protein, leading to the activation of mTORC1 that mediates the metabolic switch from fatty acid oxidation to fatty acid synthesis in ALD [4]. Previous work from our lab demonstrated that chronic alcohol consumption increases FFA flux from blood to the liver and accumulation of hepatic FFAs, rather than TG-enriched lipid droplet, exacerbated ER stress and inflammation, which further suppressed LAMP2 and autophagy [8,9]. Several research groups also reported that FFAs activated mTORC1 and induced inflammation and cell injury in various cell types [13,15,26,27]. Considering that FFA is a nutrient-related molecule, we speculated that alcohol-induced hepatic accumulation of FFAs activates mTORC1 signaling, which leads to ER stress, LAMP2 suppression, and autophagy dysfunction. Indeed, in the current study, we found that PA treatment led to the activation of mTORC1, which then induced ER stress which resulted in subsequent reduction of LAMP2 protein and blockage of autophagy flux. Suppressing mTORC1 activation by rapamycin attenuated PA-induced ER stress, restored LAMP2 protein, and improved autophagy flux. Our in vivo study further confirmed that rapamycin-mediated inhibition of mTORC1 signaling reduced ER stress, restored LAMP2 protein, and reversed alcohol-induced liver injury.

CHOP is a downstream target of ATF4 which is involved in ER stress-induced cell injury and death. Our recent publication has shown that FFA-mediated induction of ATF4 leads to increased CHOP protein and decreased LAMP2 protein, while ATF4 knockdown suppressed CHOP accumulation and restored LAMP2 protein abundance [9]. However, it is not clear if CHOP is the downstream mediator of ATF4 that causes the suppression of LAMP2 or if changes in the protein levels of CHOP and LAMP2 are parallel events both downstream of ATF4. To answer this question, we created a stable CHOP knockdown cell line and treated cells with PA. We demonstrated that knockdown of CHOP partially restored PA-mediated LAMP2 reduction, while p-S6 and ATF4 protein levels were not significantly impacted, indicating that CHOP is an ATF4 downstream mediator, and mTORC1-acticated ATF-CHOP signaling negatively regulates LAMP2 under FFA-induced lipotoxicity.

The levels of LC3II were once considered a gold standard for investigating autophagy. However, recent studies have pointed out that this method has limitations, as LC3II accumulation may represent either blockage of autophagy pathway or induction of autophagy activity [28,29]. In our previous study, we consistently observed that LAMP2 protein was suppressed and LC3II protein was accumulated due to fatty acid-induced ER stress while attenuation of ER stress resulted in the restoration of LAMP2 protein level and normalization of LC3II accumulation in cell and animal models of ALD [9]. In this study, the expression pattern of LC3II from the animal model supplemented with rapamycin yielded a similar result. Nevertheless, while addition of rapamycin to PA-treated cells largely reduced ER stress and restored LAMP2 protein abundance, the levels of LC3II protein, unexpectedly, further increased. Because rapamycin is known to induce autophagy activity [30], these observations prompted us to examine autophagy flux using mRFP-GFP tandem fluorescent-tagged LC3, which is a more reliable method in determining autophagy flux because successful fusion of an autophagosome with a lysosome to form an autolysosome leads to the visualization of only RFP signal due to its resistance to acidic environment inside the autolysosome [31]. PA treatment induced a strong accumulation of both GFP and RFP signals in Hepa-1c1c7 cells, indicating disrupted fusion of autophagosome and lysosome and blockade of autophagy flux. The level of RFP signal was further induced while minimal GFP signal was observed under PA and rapamycin cotreatment. Thus, the accumulation of LC3II by rapamycin in the cell study indicates enhanced autophagy activity rather than suppression of autophagy flux.

In summary, the present study demonstrated that FFAs activate mTORC1-ATF4-CHOP signaling pathway, which downregulates LAMP2 and impairs autophagy flux. Inhibition of mTORC1 activation by rapamycin suppressed FFA-mediated ER stress and improved autophagy flux. Rapamycin-induced mTORC1 inactivation in mice suppressed ER stress, and restored LAMP2 and autophagy function, thereby ameliorating alcohol-induced liver injury. Activation of mTORC1 signaling and accumulation of CHOP were also detected in the liver of patients with SAH. These findings offer novel insights into the role of hepatic FFAs in the pathogenesis of ALD and may shed light on the development of therapies targeting FFA-mediated lipotoxicity via modulation of mTROC1-ATF4-CHOP4-LAMP2 pathway.

## Data Availability

The study did not generate any data other than results that are reported in the manuscript.
